# Compact SAW aerosol generator

**DOI:** 10.1007/s10544-017-0152-9

**Published:** 2017-01-27

**Authors:** A. Winkler, S. Harazim, D.J. Collins, R. Brünig, H. Schmidt, S.B. Menzel

**Affiliations:** 1 SAWLab Saxony, IFW Dresden, Helmholtzstr. 20, 01069 Dresden, Germany; 2 Singapore University of Technology, Engineering Product Design, 8 Somapah Road, Singapore, 487372 Singapore; 3 BelektroniG GmbH, Hauptstraße 38, 01705 Freital, Germany

**Keywords:** Surface acoustic wave, SAW, Atomization, Aerosol source, Miniaturization, Mass scale production, Microchannel, Fluid supply

## Abstract

In this work, we discuss and demonstrate the principle features of surface acoustic wave (SAW) aerosol generation, based on the properties of the fluid supply, the acoustic wave field and the acoustowetting phenomena. Furthermore, we demonstrate a compact SAW-based aerosol generator amenable to mass production fabricated using simple techniques including photolithography, computerized numerical control (CNC) milling and printed circuit board (PCB) manufacturing. Using this device, we present comprehensive experimental results exploring the complexity of the acoustic atomization process and the influence of fluid supply position and geometry, SAW power and fluid flow rate on the device functionality. These factors in turn influence the droplet size distribution, measured here, that is important for applications including liquid chromatography, pulmonary therapies, thin film deposition and olfactory displays.

**Electronic supplementary material:**

The online version of this article (doi:10.1007/s10544-017-0152-9) contains supplementary material, which is available to authorized users.

## Introduction

Aerosol generation using surface acoustic waves (SAWs) on piezoelectric chip substrates was first demonstrated by Kurosawa et al. in 1995 (Kurosawa et al., 1995a). Since then, several promising fields of application for this technique were reported, including inhalation therapy (Qi et al., 2009; Rajapaksa et al., 2014), olfactory displays (Nakamoto et al., 2014), micro- and nanoparticle synthesis (Alvarez et al., 2008; Kim et al., 2005; Qi et al., 2011), thin film deposition (Murochi et al., 2007; Darmawan et al., 2014; Winkler et al., 2016a) and mass spectroscopy of low- or non-volatile fluids (Ho et al., 2011; Huang et al., 2012; Monkkonen et al., 2016; Winkler et al., 2016b).

Until now, however, SAW atomizers are not utilized outside of laboratory settings, which can be attributed to the functional limitations of the lab setups, including issues with fluid supply and the resulting poor stability and reproducibility of the aerosol properties. In order to overcome these, we have previously demonstrated photolithographically-structured SU-8 microchannels for on-chip integrated fluid supply (Winkler et al., 2015). In comparison to conventional fluid supply methods, these microchannels permit parallel and cost-effective manufacturing of SAW atomizer chips on the wafer-scale using techniques compatible to standard CMOS technology. In addition, the utilized materials are compatible with most relevant fluids and biological applications.

In this work, we significantly expand the development of this system to permit reliable and robust operation with fast chip interchange, and examine the influence of the fluid supply position, the fluid supply geometry and the acoustic wave field, on the acoustofluidic interactions taking place at the boundary of the acoustic path and the atomization zone. Compared to other results reported in the literature, our fluid supply approach thereby allows a high level of experimental control, continuous stable device operation and unique boundary conditions enabling reproducible acoustofluidic interaction. Based on our investigations, we formulate the requirements for placement and geometry of the fluid supply for reproducible and reliable atomizer operation. Furthermore, we demonstrate a compact SAW aerosol generator suitable for mass-scale production.

## **System principles**

The existence of a fluid film in the SAW propagation path is essential for the aerosol droplet production, as fluid droplets with diameters between 0.1–100 μm are ejected from such a film (Ang et al., 2016; Collins et al., 2012; Wang et al., 2016). Commonly, the term „fluid film“ is defined as a body of fluid with a thickness that is substantially smaller than any of its horizontal dimensions. In SAW atomization, it is further defined by the existence of Rayleigh streaming as the dominant streaming mechanism within its vertical dimension, i.e. the film thickness, on the order of the acoustic wavelength in the fluid (given by λ_f_ = λ_SAW_*c_l_/c_s_, typically tens of microns for 10–100 MHz SAW). Acoustowetting is the driving force for the development of such a fluid film out of a parent liquid body positioned at the boundary of the acoustic beam and for atomization, i.e. droplet ejection, out of this fluid film. The current state of knowledge on acoustowetting is briefly described here to clarify the discussion of the made observations.

Acoustowetting is defined by a contact angle reduction and the fluid volume spreading in response to an oscillating substrate. The phenomena could be described as follows: The out-of-plane component of the SAW (u_3_) leaks pressure waves into a fluid on the chip surface. These longitudinal pressure waves can be reflected at interphases between materials with mismatched acoustic impedance, i.e. the liquid/air and the liquid/solid interphase. In the case of a stationary resonance of these wave reflections, the magnitude of the acoustic radiation pressure exerted on the liquid/air interphase, i.e. the free surface of the liquid, is reduced to a level where it may be balanced by opposing capillary stress (Altshuler & Manor, 2015; Manor et al., 2011). Stable resonance conditions exist for distinct values of film thickness, self-selected from radiation pressure minima at the free fluid surface to minimize the internal pressure (and thus energy) state. Though an acoustowetting fluid film can transiently assume several different local pressure minima, the specific fluid parameters, especially its viscosity and surface tension, as well as the geometric boundary conditions of the fluid source, i.e. the microchannel outlet, determine which local pressure minimum is the global one. Fluid viscosity in part determines the acoustic attenuation length and the viscous stress at the interface, and thus the strength and scale of acoustic streaming, i.e. the viscous boundary flow and associated convective contributions (Stokes drift, Rayleigh streaming) as well as a directional drift imposed by travelling SAW components. Therefore, viscosity may play a significant role not only in the atomization process, but also in the stabilization of the film height and convective transport leading to film spreading. Moreover, the existence of an acoustowetting fluid film in the first instance is subject to contact angle constraints. When Rayleigh streaming is the dominant streaming mode, the fluid height near the contact line should be within a distance on the order of the wavelength of the longitudinal wave from the substrate, requiring an intermediate contact angle (Alvarez et al., 2008; Rezk et al., 2012a; Kurosawa et al., 1997). Too high a contact angle, and streaming on the scale of multiple wavelengths (Eckart streaming) in a fluid bulk results (Alghane et al., 2011; Destgeer et al., 2016), without fluid film formation. Our investigations show that the contact angle should be below the Rayleigh angle θ_R_, determined by the speed of sound of the substrate and the fluid, for fast resonance stabilization. However, very low contact angles θ_C_ ≲ 10° hinder precise fluid supply, as the fluid easily spreads over the entire chip surface.

An interesting characteristic of an acoustowetting fluid film is that it can advance along SAW displacement gradients, moving from regions of low SAW amplitude to regions of high amplitude. This film propagation makes it possible to separate the locations of fluid supply and atomization zone, as it is the case for a fluid supply at the acoustic beam boundary used in this study.

In addition, the existence of a sub-micron thin film dominated by viscous boundary layer flow has been demonstrated. It is, however, highly unlikely that this (very) thin film is a relevant source of atomized droplets, if only because of the fractional volume of this region.

## Experimental

### Description of individual components

**Fig. 1:**
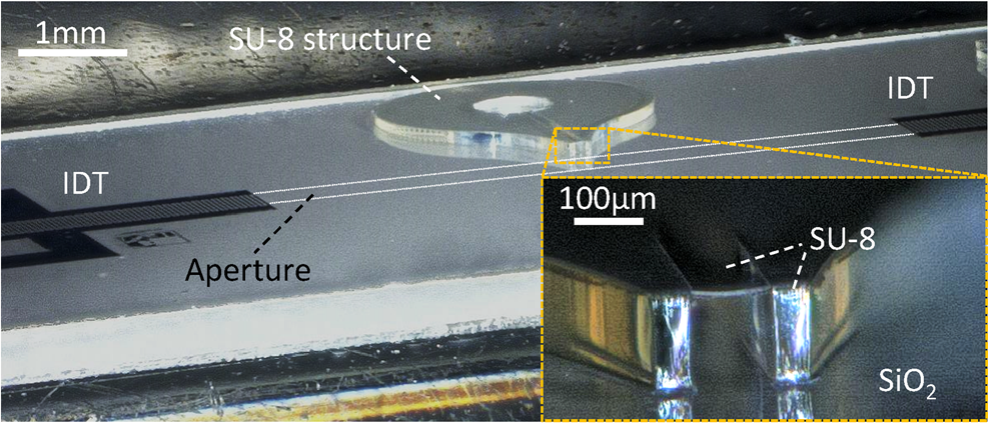
Tilted photomicrograph of a SAW chip with two IDTs and SU-8 microchannel structure (IDT aperture indicated by dotted white lines); inset shows magnified microchannel outlet

**Fig. 2:**
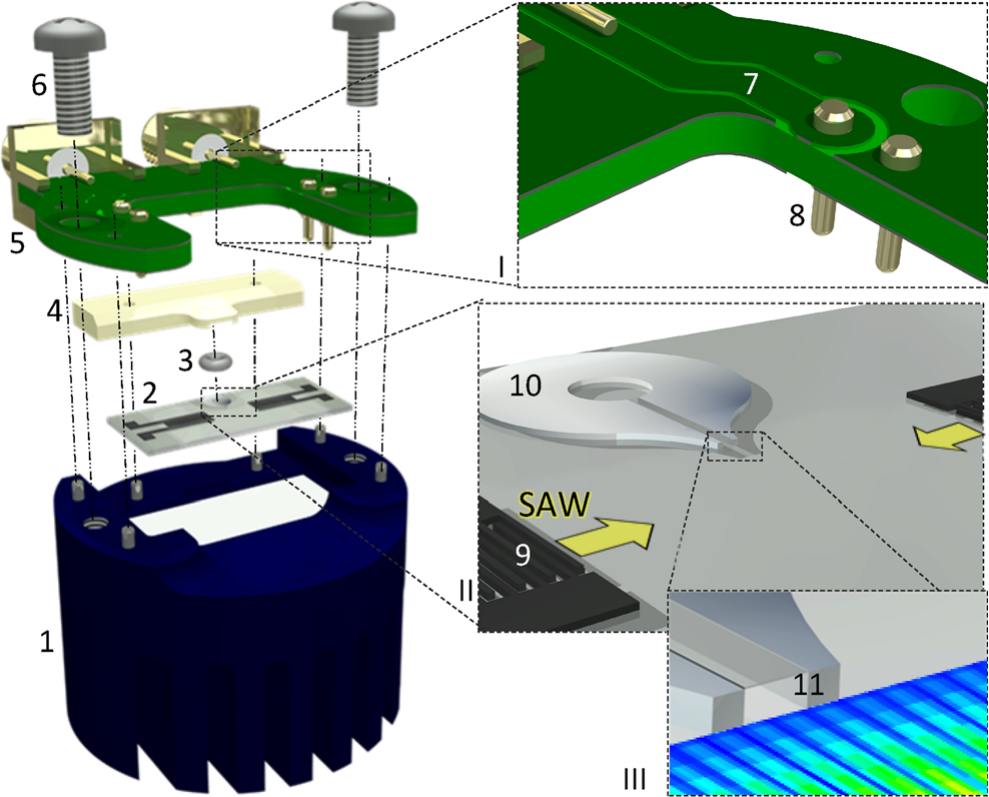
Assembly sketch of the compact SAW aerosol generator with its individual components: 1 = chip holder with heat conductive foil (white plane), 2 = SAW chip, 3 = O-ring, 4 = fluid block, 5 = PCB, 6 = screw; Insets magnify: I) PCB with 7 = strip line and 8 = spring pins; II) SAW chip with 9 = IDT and 10 = SU-8 structure (SAW propagation directions indicated); III) partial SU-8 structure with 11 = channel outlet and indicated, measured sSAW amplitude distribution (u_3,max_) at the acoustic boundary positioned in respect to the channel outlet

The compact SAW aerosol generator setup demonstrated in this work consists of the SAW chip (Fig. 1), a heat conductive foil, a chip holder, a fluid block and a printed circuit board (PCB), described in detail below (Fig. 2).

The key component of the setup is the SAW chip, comprising a piezoelectric substrate, interdigital transducers (IDTs) and the polymer microchannel/s, all produced by conventional lift-off photolithography. On the chips used in this study (128°YX-LiNbO_3_, 8 mm × 19 mm chip size), pairs of IDTs (λ/4, λ = 90 μm, 46 finger pairs, 0.5 mm aperture, f_0_ ≅ 42.9 MHz) matched to 50 Ω impedance by design and consisting of subsequent layers of Ti (5 nm) and highly-textured Al (295 nm) were prepared via electron-beam evaporation and lift-off technique. For standing SAW (sSAW) excitation, the two IDTs oppose each other with a separation of 6 mm. A 1000 nm thick SiO_2_ layer (Winkler et al., 2009) was sputter-deposited on the chip surface in order to inhibit aluminum corrosion and to establish a chemically compatible surface material on the piezoelectric substrate. Subsequently, SU-8-50 photoresist (Microchem Corp.) was spin coated on each chip surface, forming an approximately 100 to 140 μm thick layer. The SU-8 was structured via double-exposure photolithography (details described elsewhere (Winkler et al., 2015)). The general chip layout, i.e. the placement, number and properties of IDTs and SU-8 structures are subject of optimization for a given aerosol generation task. As will be shown, the placement of the channel outlet and its geometrical properties, in respect to the SAW field generated by the two IDTs, have significant influence on the device functionality.

A CNC milled and anodized aluminum platform was prepared as chip holder, comprising cooling fins on its bottom side for efficient heat dissipation, a cavity on the top side for placement of heat-conductive foil and the SAW chip. While thermal control is desirous for SAW atomization generally, where lower operating temperatures promote longer device lifetimes, cooling fins are especially necessary for the testing under laboratory conditions, as constant aerosol production cannot be guaranteed for different experimental parameter sets, e.g. used fluids or fluid flow rates. If no fluid is supplied while the SAW is excited and the resulting heat is not dissipated, the device temperature can increase and chip damage can occur. However, as efficient atomization to our observations causes no excessive heating this component could be minimized or eliminated in the future in a specific application targeted setup. The chip holder also comprises milled guidance features and stainless steel guidance pins for the heat conductive foil (TGF-Z0500-NS, Hala Contec GmbH & Co. KG), the SAW chip, the fluid block and the PCB. The fluid block – in a first and very simple design – contains a fitting for an O-ring (EPDM 70 Shore A, 0.74 mm × 1.02 mm, Kremer GmbH, Germany) and a drilled hole for the connection to an external fluid source via a stainless-steel dosage tip. The O-ring seals the fluidic connection by directly connecting to the SU-8 structures without damaging them.

In this work, the fluid flow rate was maintained constant up to 1000 μl/min by a neMESYS laboratory syringe pump (Cetoni GmbH, Germany) for maximum control and experimental flexibility. However, the integration of miniaturized pumps in a SAW atomizer system is possible when the parameter space is defined for a certain application and was already successfully demonstrated in the literature (Ariyakul & Nakamoto, 2014). Deionized water, ethanol and an aqueous 50%v Glycerol solution were used as model fluids. The customized PCB (manufactured by Würth Electronic GmbH & Co. KG., Germany) comprises strip lines matched to 50 Ω impedance, female SMA-PCB connectors and gold-coated spring pins, which directly contact the pads on the chip surface. For radio-frequency (rf) compatibility, several vias connecting the front-side and backside ground plane were introduced. The integration of additional electronic elements or logic circuits is easily possible, but was not part of this study. The PCB electrically contacts and mechanically retains the chip and the heat conductive foil underneath. Two screws are used to interconnect the assembly.

To maintain experimental flexibility in our setup, RF signals were supplied at the working frequency of the transducers, i.e. at minimum power reflection, via SMA cables from a dual-channel Power SAW F20 signal source (BelektroniG GmbH, Germany) with a load power of 1.5 to 3.5 W supplied to each IDT. The electronics design of a small and portable rf signal generator (e.g. (Qi et al., 2009)) was not an intended part of this study, but can be achieved by tailoring to a desired aerosol generation task with narrowed range of aerosol and SAW parameters.

### Assembly of the compact SAW aerosol generator

Figure 2 indicates the assembly steps of the compact SAW aerosol generator, described briefly as follows. In preparation of each experiment, the SAW chip with the heat-conductive foil on its backside and the fluid block with the O-ring are subsequently positioned on the chip base in a plug-and-play fashion. Thereby, the vertical features of chip base and fluid block are designed in order to ensure a defined positioning and distance of fluid block to chip and PCB to chip. By mounting the PCB and fixing it via screws in the last step, the fluid block is pressed downward, deforming the O-ring and ensuring a reliable fluidic connection. At the same time, the spring-pins contact the bond pads and retain the chip mechanically with defined force (here, 80 cN for each spring pin). This straightforward approach allows the concurrent realization of fluidic, mechanic and electric connections. The accuracy of the fluid supply placement is therefore determined by the chip layout only, i.e. small variations in the O-ring and fluid block positioning do not alter the channel outlet position with respect to the SAW field, an important factor for reproducible aerosol generation. Due to the high mechanical stability of the SU-8, the thin channel coverlid withstands the pressure of the O-ring and that caused by high flowrates (> 1 ml/min) even after several assembly/disassembly steps.

### Further experimental conditions

Wave field measurements were carried out using a UHF 120 Laser Doppler vibrometer (Polytec GmbH, Germany). In order to avoid acoustic reflections, high viscosity photoresist was applied in front of the chip edges for these measurements. Video recordings of the whole aerosol generator were carried out using a α5000 camera equipped with an SEL-55210 objective (Sony Europe Ltd.). Video micrographs were recorded using a SMZ-2 T stereomicroscope (Nikon Co.) with attached TSOUDC3V USB camera (Thalheim Spezialoptik GmbH, Germany). The aerosol droplet size distributions of atomized DI-water were investigated using a Helos KR laser diffractometer (Sympatec GmbH, Germany) in the range of 1 to 100 μm. The aerosol beam was measured for 3 × 15 s in a distance of 20 mm to the chip surface with a laser spot of approximately 20 mm diameter. A suction device was used to collect the aerosol above the measurement spot in order to prohibit fluid condensation on the diffractometer lenses. Droplet sizes were calculated using the Mie scattering theory. Individual peaks almost ideally follow logarithmic normal distribution functions, which were fitted using the parameters of peak area, arithmetic mean E(x) and arithmetic standard deviation SD(x).

## Results and discussion

### Wave field and resulting SAW chip requirements

**Fig. 3:**
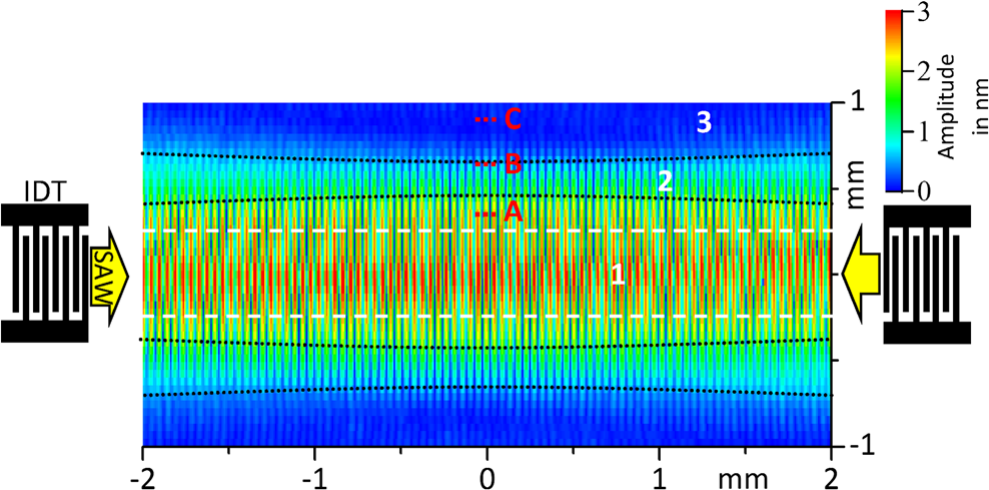
Typical sSAW amplitude distribution (out of plane amplitude û_3_) between two IDTs (λ = 90 μm, w = 500 μm, w/λ = P_Load_ = 200 mW, 533 × 76 measurement points); IDT positions, important regions of the wave field (1, 2, 3) and their arbitrarily chosen boundaries (black dotted lines), the IDT aperture boundaries (white dashed lines) and employed channel outlet positions (A, B, C) indicated

The acoustic wave field, i.e. the distribution of the out of plane amplitude u_3_ and the phase (i.e.), determines the physical conditions at the fluid supply position, at the atomization zone and in the interconnecting fluid film. As the basic features of any standing SAW field are comparable, they are briefly discussed here, based on Laser-Doppler vibrometry measurements of the devices used in this study. Fig. 3 shows the measured maximum amplitude distribution of the Rayleigh-type sSAW (surface-normal (û_3_) component distribution only) excited between the two interdigital transducers (IDTs) on the SAW chip. In supplementary video 1, the momentary vertical displacement is shown.

Here, the nodes and antinodes of the standing SAW field caused by interference of counter-propagating SAWs are clearly identifiable. Furthermore, diffraction, caused by the finite IDT aperture and the anisotropic substrate properties, was found to result in three identifiable regions: Region 1, i.e. the center of the acoustic beam, is characterized by a high SAW displacement amplitude and amplitude variation due to diffraction, leading to an amplitude maximum. Depending on the IDT configuration used, several amplitude maxima across the beam aperture are possible (Rezk et al., 2012b). Region 2, i.e. the boundary of the acoustic beam, is determined by low SAW amplitude and an amplitude increase (positive gradient) in the direction towards region 1. Outside of these regions, a region 3 exists on the chip surface, where no measurable substrate displacement occurs. The precise positions of the boundaries between these regions are subject of ongoing investigation, though are ultimately determined by diffractive effects within each IDT. Regardless, every conventional IDT configuration will excite displacement fields qualitatively similar to that in Fig. 3. The diffraction and, therefore, the dimensions of region 1 and 2 strongly depend on the substrate and IDT material, the IDT aperture, the SAW wavelength, the number of IDT finger pairs, and the orientation of the IDT with respect to the crystal orientation of the substrate (Szabo & Slobodnik, 1973; Holm et al., 1996). Special IDTs including focused, slanted-finger or chirped designs can influence the shape of the wave field; though will produce comparable diffraction and boundary features. When devices with more than one IDT are applied, the wave fields of the individual IDTs are superimposed.

In SAW fluid atomization setups, the fluid source is conventionally placed in the center of the acoustic beam (i.e. in region 1 in Fig. 3 – see e.g. (Chono et al., 2004; Ju et al., 2008; Qi & Yeo, 2008)). This may be attributed to the fact that fluid atomization requires high amplitudes to break up individual droplets from the fluid film. However, secondary effects such as fluid accumulation on the chip surface, Eckart streaming and jetting can arise if a „fluid volume“, i.e. a fluid geometry much larger than the acoustic wavelength, is present in region 1, especially if the fluid flow supply rate is not controlled. Additionally, any object placed in region 1, such as a tissue or a capillary used for fluid supply, can interact with the SAW in an undesired manner. Here, the placement of the object with respect to the wave field can then result in the appearance of wave scattering and interference at the object boundaries, high local mechanical stress and – if the material absorbs larger portions of acoustic energy – high local thermal stress. It is therefore impractical to use certain materials in the center of the acoustic beam, as local heat and pressures can lead to deleterious effects including a change in material properties, movement, and detachment or even melting/disintegration. The practical applicability of systems where the fluid is supplied in the center of the SAW propagation path (region 1), as has been the case with virtually every SAW atomization study to date, is thus limited to cases where the materials and designs can cope with these continued stresses or the atomization is limited in duration.

Summarizing the wave field properties, the underlying physics of acoustowetting known so far and our own observations, we suggest an ideal, continuously driven SAW atomization chip to fulfil several criteria:

1. An on-chip fluid supply (for high accuracy, reproducibility and mass-manufacturability)
2. Positioning of the fluid supply at the boundary of the acoustic path (for spatial separation of fluid supply position and atomization position, minimized interaction of the means of fluid supply with the SAW and continuous atomization off a fluid film),
3. Accuracy of the fluid supply geometry and placement well below the SAW wavelength (for reproducible SAW-fluid interaction),
4. Tailored design of the IDTs and the resulting SAW field to fulfil the fluid supply and atomization needs (for optimal utilization of the SAW power and minimized secondary effects, including device heating, Eckart streaming and jetting), and
5. Biological and chemical compatibility of all implemented materials to the used fluid solutions.

As a further development of the capillary slit fluid supply (Kurosawa et al., 1995a; Kurosawa et al., 1995b; Soluch & Wrobel, 2003; Soluch & Wrobel, 2006), a fluid supply via SU-8 microchannels placed at the boundary of the acoustic propagation path (region 2) has recently been demonstrated (Winkler et al., 2015). Due to the use of photolithography for the channel structuring, the position of the fluid meniscus as well as its height and width can be tailored with sub-micron resolution, well below the SAW wavelength. When fluid supply and the SAW field are appropriately positioned, the SAW interacts with the fluid directly at the channel outlet, forming a fluid film by longitudinal wave resonance at this position. Thereby, the boundary conditions of height and width of the channel outlet are expected to contribute to fluid film formation and to determine the stable longitudinal wave resonance condition in the formed fluid film under SAW influence.

Furthermore, the microchannel structures can be designed in a way that ensures a sufficient mechanical stability of the channel walls and at the same time minimizes the effective polymer cross section in the acoustic beam boundary to minimize the SAW-SU-8 interaction and associated acoustic energy uptake. Due to these reasons, we employed an SU-8 microchannel fluid supply in this study.

### Aerosol generation and crucial process parameters

The behavior of the compact SAW aerosol generator was investigated for different SU-8 channel placement conditions, model fluids, SAW power levels and fluid flow rates. Additionally, the turn-on/−off behavior was studied. The observations made are summarized here.

**Fig 4:**
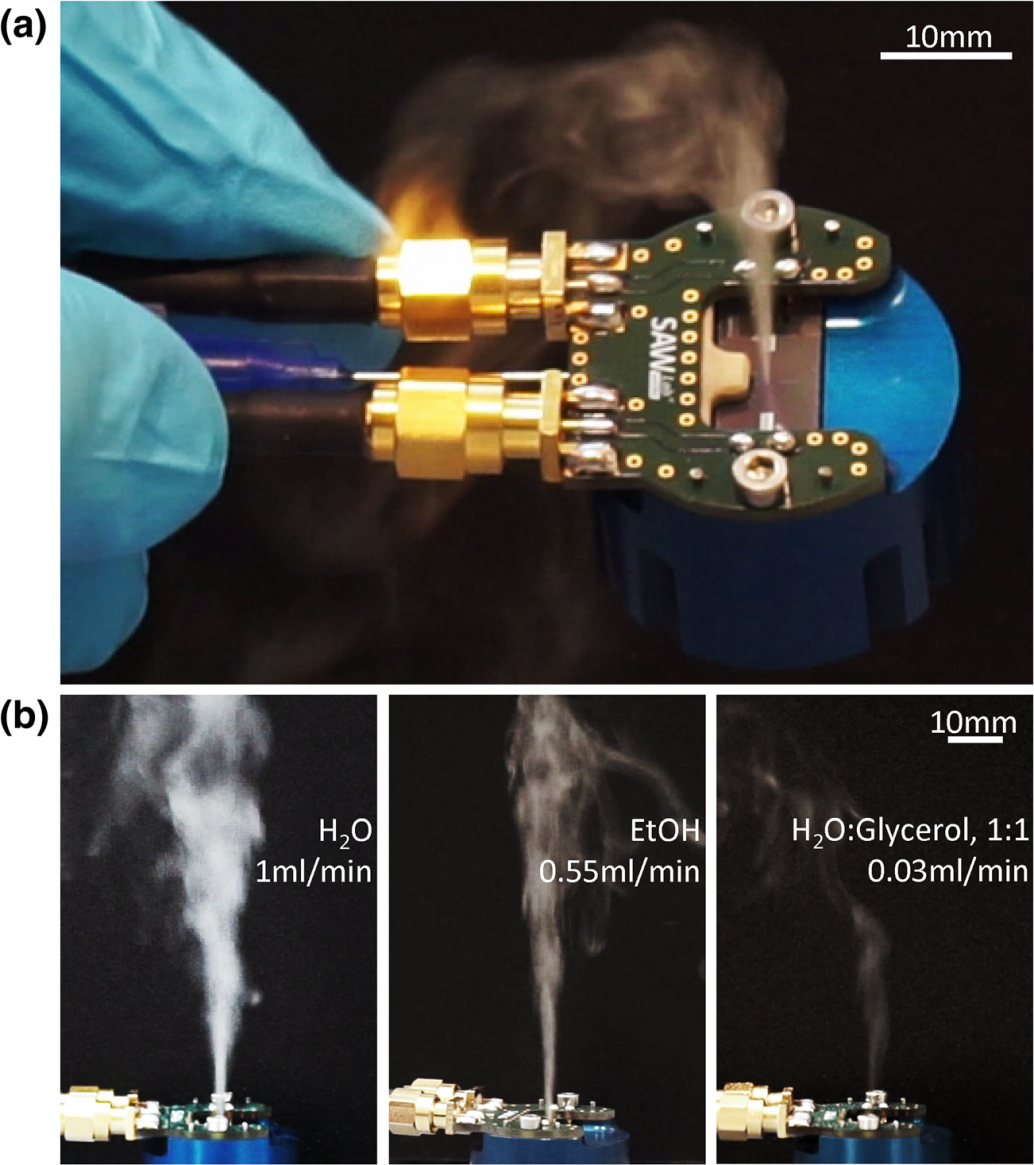
Aerosol generation using the compact sSAW aerosol generator: **a** view on tilted setup during Ethanol atomization (140 μl/min), **b** Comparison of the aerosol beam for three different fluids at the maximum possible flow rate for a given setup and constant SAW parameters: DI-Water (1 ml/min), Ethanol (0.55 ml/min) and 50%v aqueous Glycerol solution (0.03 ml/min)

Subsequently after SAW excitation and concurrent fluid supply, SAW-fluid interaction and associated acoustowetting lead to the extension of a fluid film from the microchannel outlet in region 2 towards the atomization zone in region 1 (as in Fig. 3), where the SAW has a sufficiently high amplitude to eject droplets out of the fluid film. Effects due to gravity are negligible in describing the behavior of this small amount of fluid on the chip surface. Figure 4a shows the aerosol generator during the atomization of ethanol with a flow rate of approx. 140 μl/min. There is no change in the aerosol plume geometry or the atomization behavior, even when the setup is tilted or turned upside down (supplementary video 2).

Experiments with different fluids (Fig. 4b) show a limitation of the maximum realizable flow rate and the aerosol beam height by the fluid properties for otherwise constant experimental boundary conditions (supplementary video 3). While the exact physics of the aerosol generation remain unclear, the dynamic viscosity, surface tension, density and electric conductivity of the fluid as well as the contact angle to the substrate may be responsible, as they may influence the acoustowetting, the longitudinal wave velocity/propagation angle/attenuation and the droplet ejection mechanism.

**Fig 5:**
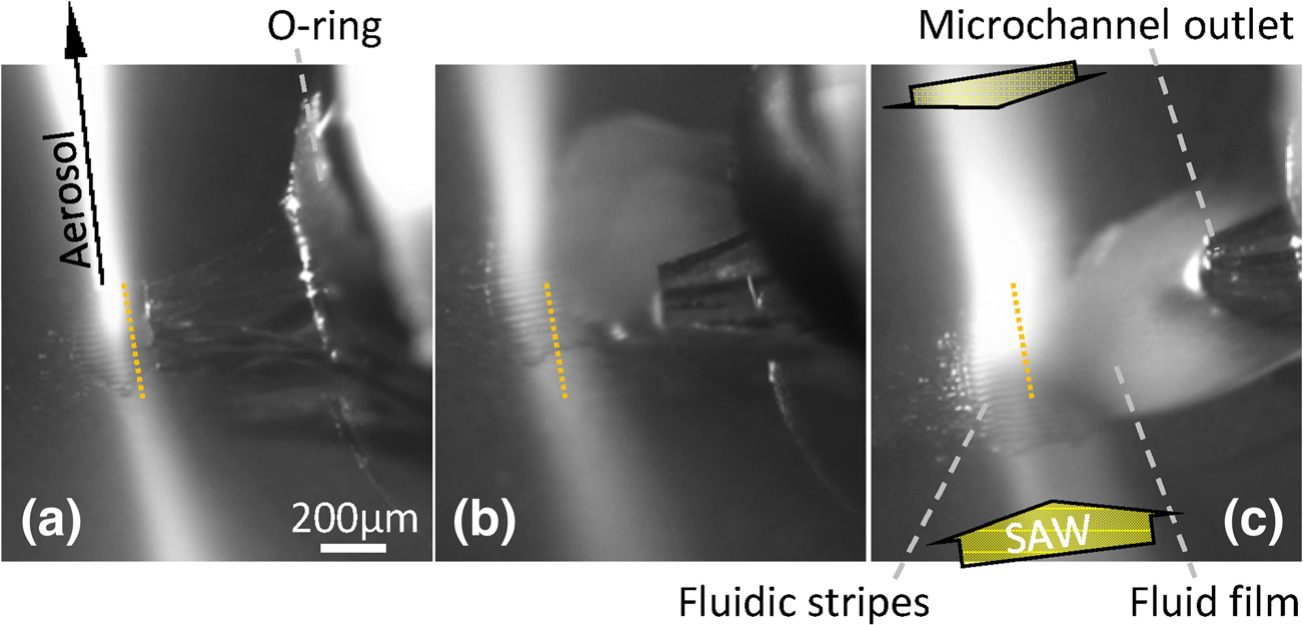
Comparison of three different fluid supply positions; distance measured from the boundary of the IDT aperture: Position **a** 100 μm (region 1), **b** 400 μm (region 2–3) and **c** 650 μm (region 3); Positions indicated in measured SAW field (Fig. 3); boundary of the IDT aperture close to the channel outlet (dotted yellow lines) indicated

**Fig 6:**
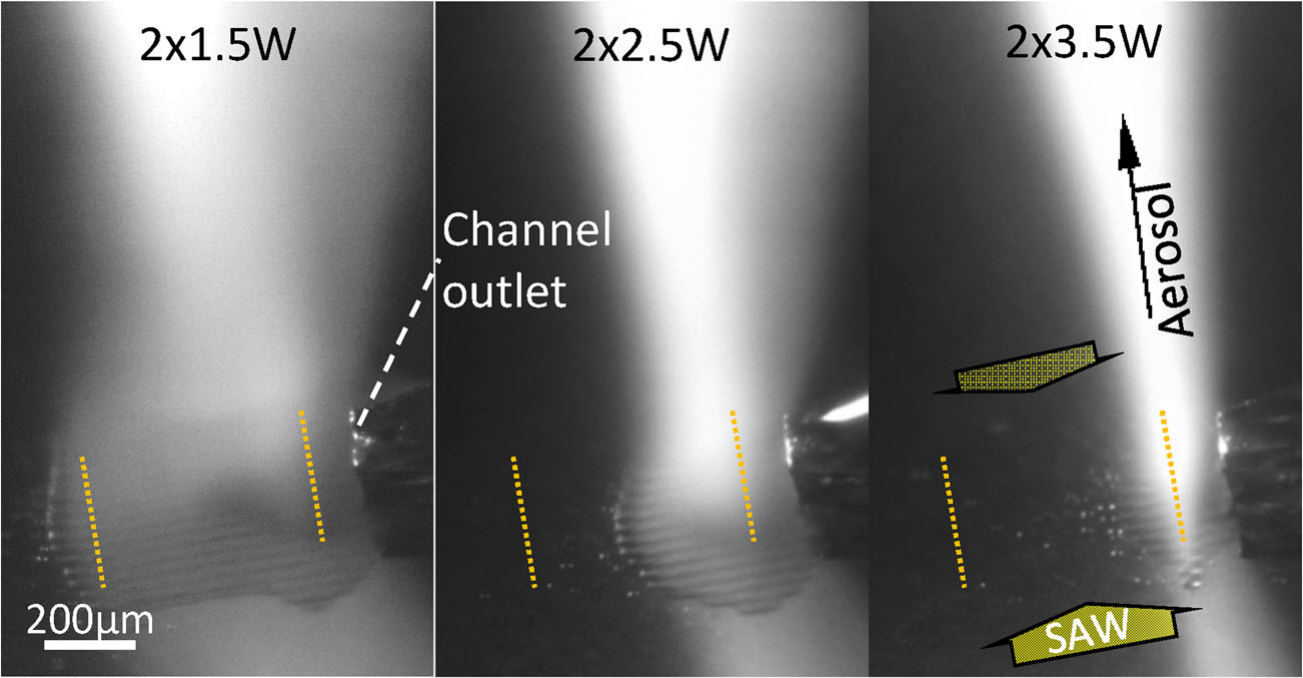
Micrographs of the SAW chip for continuous fluid supply (100 μl/min) with different sSAW power (2 × 1.5…3.5 W) at improved fluid supply position (i.e. Pos. A in Figs. 3 and 5); boundaries of the IDT aperture (dotted yellow lines) and SAW propagation directions indicated

In general, the observation of the atomization zone with high resolution renders visible several features of the acoustofluidic interaction (Figs. 5 and 6, supplementary video 4-6): The aerosol plume emanates from the acoustically stabilized fluid film at the atomization zone, i.e. a wave field region with sufficiently high SAW amplitude. Due to the finite camera exposure length, the aerosol and the fluid film, both of which are driven by perturbations with timescales in the nanoseconds-range, can only be recorded with blurry boundaries. In addition, a film region with a topography resembling the local sSAW amplitude distribution is visible, whereby a separation of individual fluidic stripes can be measured corresponding to λ_SAW_/2 or 45 μm for the 90 μm wavelength devices used here (compare to wave field in Fig. 3). This film shaping is driven by a balance between acoustic radiation pressure and capillary stress (Manor et al., 2015; Scortesse et al., 2002; Biwersi et al., 2000) in a region with reduced fluid volume.

In order to evaluate the influence of the channel outlet position on the atomization process, the distance between the outlet and the aperture boundary was varied from approx. 100 to 650 μm (Fig. 5 and supplementary video 4). Atomization was carried out at a constant fluid flow rate of 100 μl/min and a SAW power of 2 × 3 W. When the channel outlet is positioned close to the beam boundary (e.g. position A), constant and highly stable aerosol generation can be achieved. However, when the distance to the aperture center is too small, the high SAW amplitude may lead to damage the microchannel material due to local acousto-viscous heating. With increasing distance, the length of the fluid film interconnecting fluid supply and atomization zone increases, while the location of the atomization zone stays more or less constant. With increasing distance, atomization increasingly turns unstable, as the local wetting conditions and capillary stresses become more important to defining the fluid shape (e.g. position B). If the distance is increased further, atomization turns discontinuously (e.g. position C): The supplied fluid accumulates in form of a fluid volume at the microchannel outlet until its contact line reaches the acoustic beam boundary. Then, in short time, the droplet is thinned by acoustowetting as well as fluid jetting, and atomization occurs until the fluid feed is interrupted by retraction of the parental droplet. This dependence of the atomization behavior on the channel outlet position leads to an optimization problem in the device design. To achieve a stable and reproducible atomization, further investigations on the influence of the SAW power, the flowrate and the turn-on/off behavior were carried out in a setup with a reduced separation of 100 μm from channel outlet to aperture center (position A).

To investigate the influence of the SAW power, the flow rate was maintained constant at 100 μl/min while the power was varied. Optical micrographs of the SAW chip during atomization are shown in Fig. 6 and supplementary video 5. These demonstrate that the fluid film extension significantly depends on the applied SAW power. Interestingly, the fluid-covered area decreases in size with increasing SAW power. This can be explained taking into account the local SAW amplitude in respect to the channel outlet position. Higher SAW power leads to an overall increase in SAW amplitude in the acoustic beam. As a certain amplitude threshold has to be reached in order to start atomization, the position of that threshold amplitude and, thus, the atomization zone moves closer to the channel outlet for increased SAW power. As the fluid film properties depend on the local conditions at the atomization zone, also the aerosol properties including the droplet size are influenced by the SAW power (Collins et al., 2012; Bennes et al., 2005). Additionally, the wave field regions not covered by a fluid film do not support fluid atomization and the excess SAW power may promote parasitic heating.

**Fig. 7:**
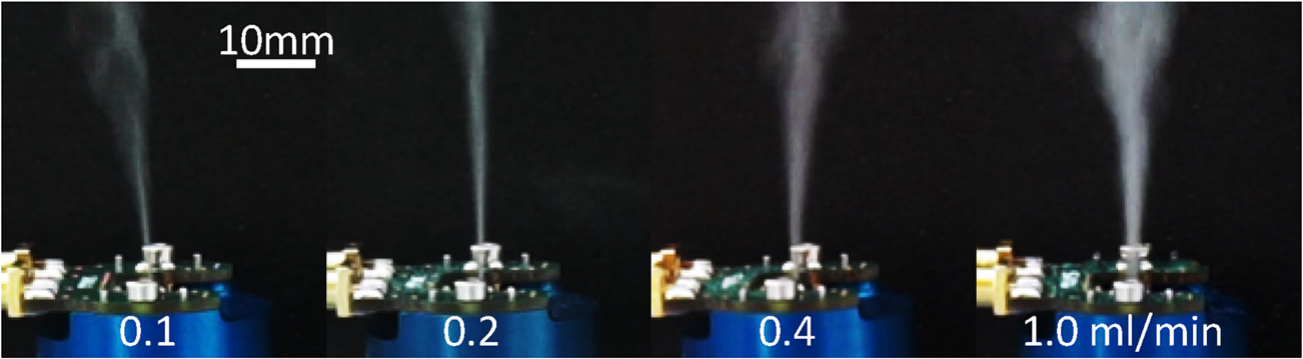
Side view on the aerosol beam for different fluid flow rates (DI-water); differences in the aerosol beam angle caused by convection of the surrounding air

Comparable behavior of the fluid film is observed, when the fluid flow rate is changed at a given SAW power. For decreasing fluid flow rate, the fluid covers a smaller area and the atomization zone moves closer to the channel outlet. The changed film conditions for different flow rate may be an explanation for the change of droplet size distribution with flow rate as reported previously (Winkler et al., 2015). Macroscopically, an increase of the fluid flow rate leads to a higher optical density of the aerosol. Figure 7 shows the aerosol beam for a water flow rate of 0.1 to 1 ml/min. We highlight that SAW fluid atomization with such a high a flow rate has not been previously reported. The maximum flow rate for a given system, however, will ultimately depend on the precise dimensions of the channel and the fluid supply position.

**Fig. 8:**
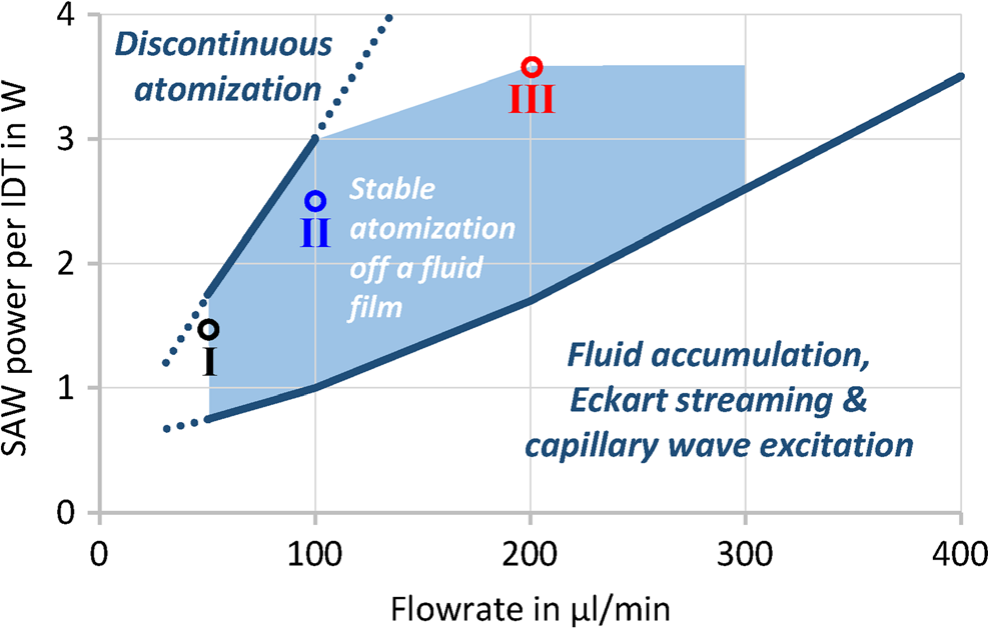
Atomization regimes observed for a 90 μm sSAW chip with improved fluid (DI water) supply position (i.e. Pos. A (Figs. 3 and 5)); Power-flowrate combinations for droplet size measurements indicated (I-III)

We observe that fluid flow rate and SAW power can be adjusted separately while maintaining stable atomization. Figure 8 shows the conceptual atomization regimes determined for the device used in this study. We find that limitations arise due to the applied power and flow rate; low flow rates will render the atomization process discontinuous as the atomization rate exceeds the rate of fluid supplied to the chip. For moderate fluid flow rates, the amount of fluid on the chip surface fulfils the generation of a fluid film, and atomization occurs continuously if sufficient power is applied. At drastically increased flowrates for a given power, however, the rate of fluid supplied to the chip surface can exceed the atomization rate, leading to the stop of atomization and fluid accumulation, with Eckart streaming and capillary wave excitation in an expanding drop rather than film formation.

Very high SAW power may lead to local temperature increase and device damage, especially if the atomization is carried out in the discontinuous regime. Fluid coverage along the complete width of the acoustic beam (2 × 1.5 W in Fig. 6) can be seen as optimal, as otherwise portions of the wave field are not involved in the droplet generation and the unused SAW power accounts e.g. for device heating or parasitic wave mode excitation.

**Fig. 9:**
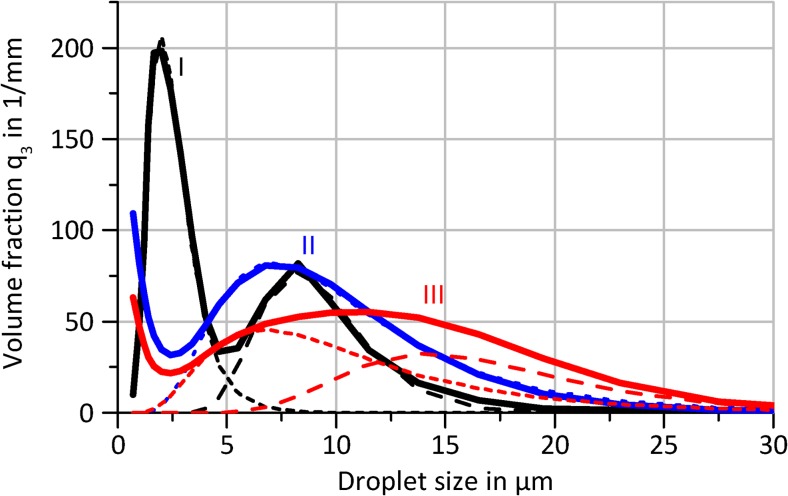
Measured droplet size distributions (volume fraction q_3_, averages of 3x15s measurements) for three chosen combinations of flow rate and power (Fig. 8); logarithmic normal distribution functions fitted to individual peaks indicated by dashed lines (the addition of individual fitted functions results in almost ideal reproduction of the measured curve)

Droplet measurements in Fig. 9 were performed for the three representative points (I-III) in Fig. 8 were carried out to quantify the relationship between an increasing atomization rate and its effect on the resultant droplet size distributions. In general, a multimodal distribution with individual peaks following a logarithmic normal distribution was observed, where the number and fraction of the peaks vary with atomization rate. The standard deviation (Var(x)^1/2^) of an individual peak amounts approx. 40…75% of its arithmetic mean E(x).

In each measurement, a fraction of very small droplets (Ø < 5 μm) was observed, comparable to those shown in previous studies (Winkler et al., 2016a; Winkler et al., 2015; Collins et al., 2012). Although no precise parameters could be extracted due to the limited measurement range of the diffractometer (Ø_min_ = 1 μm), the droplet size in this fraction generally decreases with increasing atomization rate. The origin of this droplet fraction is unclear. However, for an atomization with the device used in this study, the bulk of the atomized volume is contained within droplets with a diameter of 5 to 40 μm with x_50,3_ values of 8.6 μm, 9.2 μm and 11.9 μm for the example cases I, II and III, respectively. Interestingly, two individual peaks were observed in this size range in some of the experiments, e.g. in case III. Individual peaks in the droplet size distribution can be an indicator of different underlying physical mechanisms. We assume, that this additional peak with E(x) = 16…33 μm ± 36% can arise in cases where a fluid accumulation on the chip surface is possible, e.g. temporarily in the regime of discontinuous atomization or continuously at higher flowrates (≥ 200 μl/min). In general, no droplets larger than 80 μm were observed.

Regarding the turn-on behavior, a two-step process was observed. After the application of SAW atomization starts immediately, i.e. after less than 80 ms, once the fluid reaches the atomization zone. Then, after a short settling time, the fluid film and the aerosol beam shape stabilizes (supplementary video 6). This settling time was found to linearly decrease with increasing SAW power, e.g. from 2.5 s (2 × 2 W) to 0.9 s (2 × 3.5 W). Our observations suggest that the stabilization time is principally defined by the height of the outlet channel. In the case of a channel outlet height larger than the height of the forming fluid film, a (concave) fluid meniscus that bridges these two heights is established inside the microchannel. This partial wetting of the inner channel walls requires time to stabilize and, in turn, this transient effect determines the atomization stability. In our case, the channel height measures approx. 140 μm, while the fluid film height is estimated to be below approx. 30 μm, according to optical micrographs. The meniscus had a length of approx. 100 to 150 μm in the channel. We expect that an adjustment of the outlet height to the fluid film height could minimize the meniscus length and, therefore, the time required to stabilize the atomization.

The turn-off behavior can be described as follows: As soon as the fluid supply to the acoustic beam stops, the atomization stops immediately. However, the self-pumping effect of the SAW (Winkler et al., 2015; Kurosawa et al., 1995b) in combination with the usage of compressible tubing can lead to a slowly decreasing fluid flow rate, even if the pump is suddenly deactivated. Therefore, it can be beneficial to apply an inverse volume flow for a short interval to achieve a defined turn off behavior of the fluid flow. A direct integration of a pump in the device (as in (Ariyakul & Nakamoto, 2014)) and the associated reduction of fluidic path length could also be beneficial.

Significant heating is observed with the application of SAW power in the absence of fluid supply. This principally occurs due to (1) Joule heating at the IDT electrodes and (2) the excitation of bulk wave modes originating from reflection of SAW at the chip edges and finger electrodes, which are transmitted to the chip holder and cause viscous heating in the heat conductive foil. No excess heat production is observed in the case of completely efficient continuous atomization. As heat production is representative of under-utilized mechanical energy, it affects the overall efficiency of the SAW atomizer and its usable lifetime. It can accordingly be optimized via the fluid flow rates and SAW powers that result in fluid coverage across the SAW beam, as has been done here, or potentially the use of phononic structures to constrain displacements to the substrate (Reboud et al., 2012).

## Conclusions

While SAW aerosol generation devices hold substantial promise for therapeutic and industrial applications, they are not yet commercially available. In this work, we discuss the requirements for a reliable fluid supply with maximum process control based on the fundamental physics of acoustowetting, the local properties of the acoustic wave field and own experimental observations using a fluid supply at the acoustic beam boundary. The fundamental effects governing SAW atomization including acoustowetting, Rayleigh and Schlichting streaming, fluid film formation/propagation and droplet production, as well as the parasitic effects of jetting and Eckart-streaming, highlight the complexity of the SAW atomization process and the issues to be solved for mass-scale production of devices. We formulate important criteria for the development of optimized SAW atomization chips, taking into account the properties of wave field, fluid supply and the fluid itself.

Furthermore, using standard techniques we successfully developed a compact SAW aerosol generator with on-chip integrated fluid supply suitable for mass-production. This setup was used in various experimental conditions to highlight the influences of fluid supply position, SAW power, fluid flowrate and fluid properties. While our setup ensures high reproducibility and reliability, it also enables experimental flexibility by simple and fast interchange of application-tailored chips. Depending on the intended task, the future use of disposable chips or chips with long lifetime is possible. Additionally, the setup is compatible to the future integration together with other microfluidic components, miniaturized fluid reservoirs/pumps and small intelligent electronics for more complex and integrated signaling and analysis.

## Electronic supplementary material

Video 1 (MP4 8886 kb)

- “Animation of measured standing SAW field”

- Standing surface acoustic wave field between two interdigital transducers (IDTs, λ = 90 μm, w = 500 μm, P = 200 mW)

- Measured using Polytec UHF 120 Laser Doppler vibrometer

- Keywords: standing SAW, standing surface acoustic wave, wave field, laser doppler vibrometry

Video 2 (MP4 5360 kb)

- “Fluid atomization independent of spatial orientation”

- Atomization of Ethanol (140 μl/min atomization rate) with compact SAW aerosol generator under changing spatial orientation

- Keywords: Compact aerosol generator, SAW, surface acoustic wave, atomization, fluid, spatial, Ethanol

Video 3 (MP4 1699 kb)

- “Atomization of different fluids”

- Atomization of Ethanol (550 μl/min atomization rate), 1:1 Glycerol:Water solution (30 μl/min atomization rate) and Water (1000 μl/min atomization rate) with compact SAW aerosol generator

- Keywords: Compact aerosol generator, SAW, surface acoustic wave, atomization, aerosol, fluids, Ethanol, Glycerol, Water

Video 4 (MP4 1751 kb)

- “Atomization zone for different channel outlet positions”

- Microscopic video of the atomization zone (water atomization, 100 μl/min) and microchannel outlet with different distance (100, 400 and 650 μm) between microchannel outlet and IDT aperture boundary

- Keywords: Compact aerosol generator, SAW, surface acoustic wave, atomization zone, chip, channel, aerosol

Video 5 (MP4 2390 kb)

- “Atomization zone for different SAW power levels”

- Microscopic video of the changing location of the atomization zone (water atomization, 100 μl/min) relative to the microchannel outlet under changing SAW power conditions (2× (1.5 to 3.5 W))

- Keywords: Compact aerosol generator, SAW, surface acoustic wave, atomization zone, chip, channel, aerosol, power

Video 6 (MP4 1530 kb)

- “Turn on/off behavior at the atomization zone”

- Microscopic video of the atomization zone showing the turn on/off behavior (water atomization, 100 μl/min, P = 2 × 2.5 W) when a microchannel fluid supply is used

- Keywords: Compact aerosol generator, SAW, surface acoustic wave, atomization zone, chip, channel, aerosol, turn on, turn off
